# Optimizing Drug Delivery to the Brain for Breast Metastasis: A Novel Method for Tumor Targeting

**DOI:** 10.7759/cureus.73598

**Published:** 2024-11-13

**Authors:** Satish Krishnamurthy, Justin Y Oh, Shruti Gautham, Jie Li, Yimin Shen

**Affiliations:** 1 Neurological Surgery, State University of New York Upstate Medical University, Syracuse, USA; 2 Radiology, Wayne State University, Detroit, USA

**Keywords:** blood-brain barrier, brain metastasis, breast cancer, hyperosmolar vehicle, intraventricular

## Abstract

Introduction: Brain metastases are difficult to treat due to the blood-brain barrier limiting the delivery of therapeutic agents to the brain effectively. Intraventricular drug delivery has not been well studied for intra-axial pathologies. However, our prior work demonstrated that intraventricular drug delivery in a hyperosmolar vehicle showed preferential accumulation of drug within breast cancer tissue compared to surrounding brain parenchyma. The focus of this study was to explore the molecular parameters of intraventricular drug administration that may optimize drug delivery to intra-axial brain metastases. Our hypothesis was that a low molecular weight drug with a high osmolarity solution would increase drug delivery to tumor tissue.

Methods: We used an intracerebral breast cancer tumor model in adult female nude rats divided into six experimental groups. We examined three iron-labeled dextran molecules (3 kD, 5 kD, and 10 kD) in 337 mOsm/L solution and three different osmolarities of delivery solution (307, 353, and 368 mOsm/L) with 10 kD dextran. 7T magnetic resonance imaging (MRI) was used to analyze dextran distribution at different time points. All animals were sacrificed after two hours, and the quantity of dextran particles was determined by histopathology.

Results: Breast cancer tumor cells were successfully implanted in all rats. The MRI quantification of dextran concentration was well corroborated by histopathology. Varying the molecular size of dextran resulted in the smallest molecule reaching peak levels in tumor tissue earlier than the larger molecules, but the larger molecules remained concentrated in tumor tissue for a longer time. Varying the osmolarity of the delivery solution resulted in the preferential accumulation of 10 kD dextran in tumor tissue except for when dextran was delivered in 368 mOsm/L solution where no preferential distribution was seen.

Conclusion: Hyperosmolar intraventricular delivery of chemotherapeutic drugs could be effective in preferentially delivering drugs to abnormal tumor tissues.

## Introduction

Metastatic central nervous system (CNS) malignancy has an estimated incidence of 200,000 cases per year with most of the primary malignancies being breast cancer, lung cancer, and melanoma [1]. Depending on the hormone receptor subtype of breast cancer, the incidence per patient-year is estimated between 5% and 13% [2]. It has also been shown that breast cancer metastasis to the brain is associated with poorer prognosis [3,4]. Metastatic brain tumors are quite difficult to treat due to the unique challenge of overcoming the blood-brain barrier (BBB) [5,6]. The literature describes both invasive and non-invasive methods to overcome the BBB [6,7]. While overcoming the BBB to allow for the passage of therapeutic drugs into the brain is a mechanistically sound concept, the pharmacodynamics and pharmacokinetics of drugs in the brain extracellular space have not been well studied. Movement of macromolecules or diffusion across the brain extracellular fluid shows that diffusion is a slow process and the concentration of the said macromolecule decreases significantly with distance traversed [8]. Also, metastatic brain tumors can be heterogenous with cystic components, heterogenous contrast enhancement, and areas of necrosis. The failure to achieve adequate drug concentration in heterogenous tumor tissue may in part explain why conventional and novel therapies that have shown in vitro efficacy have disappointing clinical results [9].

We have previously demonstrated that hyperosmolar dextran infused intraventricularly directly into the brain distributes widely within the brain parenchyma, spinal cord, and cranial and spinal nerves [10]. We have subsequently shown that a novel strategy utilizing intraventricular delivery of a drug DV1 in a hyperosmolar vehicle preferentially accumulated first in breast cancer metastatic tumors compared to the surrounding brain tissue [11]. This preferential accumulation of the drug in the tumor was effective in decreasing tumor incidence that was demonstrated in cell culture, in an intact animal [11]. These findings suggest that intraventricular hyperosmolar delivery of drugs can passively target the tumor tissues. However, the BBB is thought to exclude molecules with high molecular weight, negative charge, and low lipid solubility [12]. Efflux transporters also have been known to return macromolecules back across the BBB [13]. Furthermore, the pharmacokinetics and pharmacodynamics of intrathecal drug delivery have not been well studied. While our prior results point towards a new method for passive tumor targeting in the brain, the mechanism by which this occurs must be elucidated.

In this study, we used iron dextran molecules in a hyperosmolar vehicle injected into the ventricle of rats that were implanted with a breast cancer cell line. The objective was to determine the effects of molecular size as well as the degree of hyperosmolarity on the distribution and pharmacokinetics of dextran in brain metastases. We hypothesized that a smaller molecular size and higher osmolarity vehicle would be associated with increased drug delivery to the tumor.

## Materials and methods

Subjects

Adult female nude rats (Taconic, Rensselaer, New York, United States; body weight 220-225 g; n=30) were used. They were placed in a 12-hour light/dark cycle with food and water provided ad libitum. All animal procedures implemented were in accordance with the National Institutes of Health (NIH) guidelines for the care and use of experimental animals. This experimental protocol was approved by the Institutional Animal Care and Use Committee at the State University of New York Upstate Medical University under IACUC protocol 354.

Breast cancer cell line

MDA-MB-231 is one of the breast cancer cell lines. In this work, it was transduced with the GFP signal by using *Lentivirus*, which allowed cells to be detected [2,3]. In intracranial metastases, elevated levels of CXCL12 are found in the brain, which enables MDA-MB-231 cells to breach the brain microvascular endothelial cells [4,5]. MDA-MB-231 cells are often used to design models in cancer research [4]. In this study, the MDA-MB-231 cells were cultured and preserved at 5% CO2 and 37°C in a medium containing 10% fetal bovine serum, 100 lg/mL streptomycin, and 100 U/mL penicillin [2].

Implantation of the tumor

In a chamber connected to the vaporizer, a 3-4 minute induction of 3.5% isoflurane with oxygen was administered to anesthetize the rat. Once light anesthesia was achieved, the rat was mounted on a stereotactic frame on a warm pad at 37°C throughout the procedure. The rat's nose was covered with the nose cone attached to the vaporizer. During the surgery, the isoflurane with oxygen delivered by the vaporizer was lowered to 1.5-2.5%. The rat's head was secured using a mouth holder and ear bars designed particularly for the rat. The fur was removed, and the rat's scalp was cleaned with Betadine solution and alcohol prep three times. A 1.5 cm incision through the skin was made near the midline on the dorsal side. A burr hole of 1 mm diameter was made using a variable speed electric drill at a spot marked by the following coordinates: 3 mm midline to lateral and 0.4 mm posterior to anterior. A 27G needle with a blunt tip attached to the Hamilton syringe was used to carefully inject 3 μl of the GFP MDA-MB-231 cell solution P (5×105 cells/μl) about 5 mm deep from the surface. The needle was placed into the rat's caudate-putamen nucleus and then slowly retracted after a minute. After ensuring that no active bleeding was present, the burr hole was sealed with bone wax, and the incision was closed off using a 4-0 nylon suture. Once the rat recovered from its anesthesia, it was returned to the Division of Laboratory Animal Resources (DLAR) housing facility.

Magnetic resonance imaging (MRI) and infusion of dextran

For all the rats, an MRI was performed before and after the implantation of the tumor. MRI scans were performed on a 7T horizontal magnet (BioSpen AV4 Neo; Bruker, Billerica, Massachusetts, United States). Prior to the imaging, the rats were anesthetized with isoflurane (3% induction and 2% maintenance) with medical air (1 L/min) and scavenging (vacuum -40 mmHg) after the surgical procedure. The tracer distribution kinetics in the brain were calculated using data from the following sequences: a multi-echo (ME) spin echo for R2 (=1/T2) mapping, a ME susceptibility-weighted imaging (SWI) for susceptibility mapping (36) and R2* (=1/T2*) mapping, and a fast, dual echo 2D gradient echo sequence (15 s) dynamically repeated 80 times (20 minutes).

Experimental groups

The rats were randomly divided into six experimental groups. Group 1 (10 kD dextran, 337 mOsm/L, n=5), Group 2 (5 kD dextran, 337 mOsm/L, n=5), Group 3 (3 kD dextran, 337 mOsm/L, n=4), Group 4 (10 kD dextran, 307 mOsm/L, n=4), Group 5 (10 kD dextran, 353 mOsm/L, n=5), and Group 6 (10 kD dextran, 368 mOsm/L, n=5).

Groups 1-3 test the effects of dextran tracer particle size on passive tumor targeting, while Groups 4-6 test the effects of the osmolarity of dextran. After collecting the nine time point baseline data in the dynamic scans, the iron red fluorescently labeled dextran was injected into the left lateral ventricle using a pre-catheterized customized micro-catheter (polyethylene tube). The cerebrospinal fluid (CSF) tracer process was monitored over the course of two hours by using the same R2* and SWI mapping post-contrast agent at 30, 45, 60, 90, and 120 minutes.

MRI sequence parameters

Listed below are the MRI sequence parameters for 2D ME spin echo sequence for T2 mapping, SWI for susceptibility mapping and $R2*(=1/T2*)$ mapping, and the quick dual echo 2D gradient echo scans: for 2D ME spin echo sequence for T2 mapping, long repetition time (TR)=4000 ms for ME acquisitions for T2 calculations, 10 echo times (TEs)=15, 30, …, 150 ms with a step of 15 ms, matrix size=128×128, an in-plane resolution=0.25×0.25 mm^2^, 24 slices without gap to cover the whole brain, slice thickness=1 mm, bandwidth (BW)/pixel=260 Hz/pixel, one acquisition, and total scan time (TA)=4 m 16 s; for SWI for susceptibility mapping and $R2*(=1/T2*)$ mapping, short TR=35 ms for quick acquisition, 5 TEs=5.292, …, 25.292 ms with a step of 5 ms for T2* calculations, flip angle (FA)=12° around the Ernst angle for best signal noise ratios, field of view (FOV)=32×32×24 mm^3^ to cover the whole brain, matrix size=128×128×48, resulting in a spatial resolution of 0.25×0.25×0.5 mm^3^, BW/pixel=253 Hz/pixel, one acquisition, and TA=3 m 47 s; and for the quick dual echo 2D gradient echo scans, short TR=92 ms for quick acquisition, TEs=1.85, …, 7.2 ms for T2* calculations, FA=12°, FOV=32×32 mm^2^, matrix size=192×192, resulting in an in-plane resolution of 0.167×0.167 mm^2^, BW/pixel=510 Hz/pixel, seven slices with 2 mm thickness (0.5 mm gap), and one acquisition, 15 seconds, with 80 continuous runs (total time, 20 minutes).

MRI data processing

The formula $R2*=(1/(TE2-TE1))&times;ln{s(TE1))/s(TE2)}$ was used to calculate the effective transverse relaxation rate $R2*(=1/T2*)$ of the dual echo 2D gradient echo scans. S(t) is the signal intensity, and the echo times TE1 and TE2 are 1.85 ms and 7.2 ms, respectively. The R2 from spin echo and R2* from SWI were generated offline. R2* mapping was registered to R2 mapping to obtain the same slice thickness for analysis. R2 values are stated as mean±standard deviation (the number of pixels).

Phantom studies

**Figure 1:**
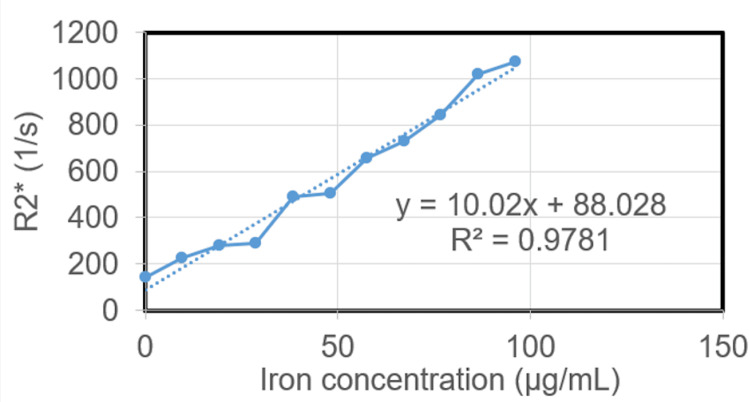
The linear relationship of R2* and iron concentration for iron dextran

**Figure 2:**
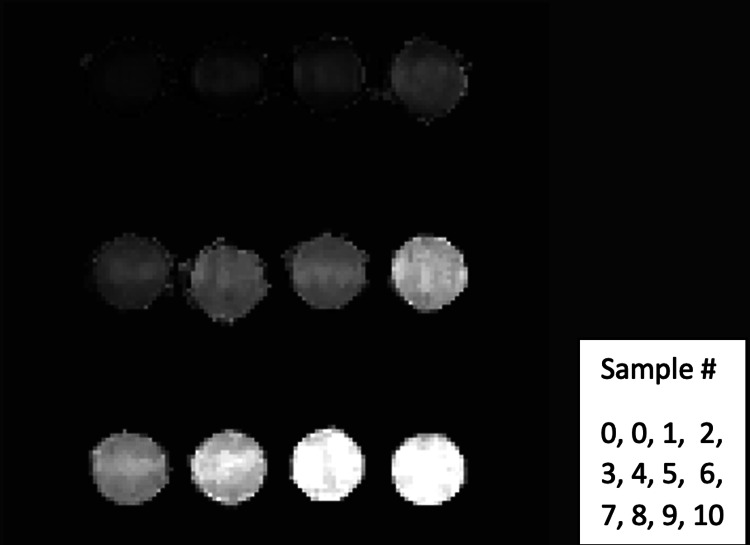
R2* mapping from MRI multi-echo gradient sequence MRI: magnetic resonance imaging

To determine the relationship between the R2* and the iron concentration of FeDextran (original solution concentration of 5 mg/mL and iron concentration of 2.4 mgFe/mL) at 7T magnet, phantom studies were performed. For sample preparations, 1.5% agarose in double distilled water was used as the diluted solution. After heating in the microwave, the agarose was completely dissolved. Iron-labeled dextran was added into the agarose solution to make different concentrations and placed in vials, which are 0.02, 0.04, 0.06, 0.08, 0.10, 0.12, 0.14, 0.16, 0.18, and 0.20 mg/ml of FeDextran (Table 1, Figure 1, and Figure 2). After the gel formed in vials, the samples were placed into a 7T scanner for MRI scans using an ME gradient echo sequence. These vials were set to be perpendicular to the main magnet field direction. The MRI parameters were as follows: TR=35 ms, eight TEs=1.6 ms, 3.1 ms, …, 12.1 ms, echo space=1.5 ms, FA=degrees, BW=1183.7125 Hz/pixel, FOV=50×50×16 mm^3^, image size=128×128×10, resolution=0.391×0.391×1.6 mm^3^, 4 averages, and TA=3 m 9 s. For each vial, R2* maps were generated using the first few echoes in which the SNR was greater than 3:1, by fitting the magnitude at different echo times to an exponential decay curve (Figure 2).

**Table 1:** The measured R2* values with various iron concentrations mg: milligram; mL: milliliter; SD: standard deviation; n: number of pixels; SE: standard error; c: iron concentration of iron-labeled dextran in solution; cFe: iron concentration of iron-labeled dextran measured by MRI; MRI: magnetic resonance imaging

Sample #	c (mg/mL)	cFe (µg/mL)	Mean±SD	n	SE
0	0	0	140.1±87.5	1303	2.423
1	0.02	9.6	228.0±53.7	920	1.769
2	0.04	19.2	279.3±65.2	2399	1.332
3	0.06	28.8	287.5±58.8	4677	0.860
4	0.08	38.4	492.7±63.3	2153	1.364
5	0.10	48	506.1±77.1	2271	1.618
6	0.12	57.6	655.7±101.2	2245	2.136
7	0.14	67.2	731.0±92.0	2128	1.995
8	0.16	76.8	846.1±161.9	2110	3.525
9	0.18	86.4	1018.3±157.8	2073	3.465
10	0.20	96	1074.0±140.5	2117	3.053

Tissue collection

At the rats' designated sacrifice times, a plane of deep anesthesia was achieved, and they were transcardially perfused with cold saline and then 4% paraformaldehyde in 0.1 M phosphate buffer with a pH of 7.4. The tissues were collected and preserved in a fixation buffer for another two hours. Then, they were transferred to a 20% sucrose in 0.1 M PB buffer in the cold room (4°C) overnight. Once frozen, the brain tissue was cut into thick slices of 20 μm using the frozen microtome. The entire brain was collected for histological analysis.

## Results

**Figure 3:**
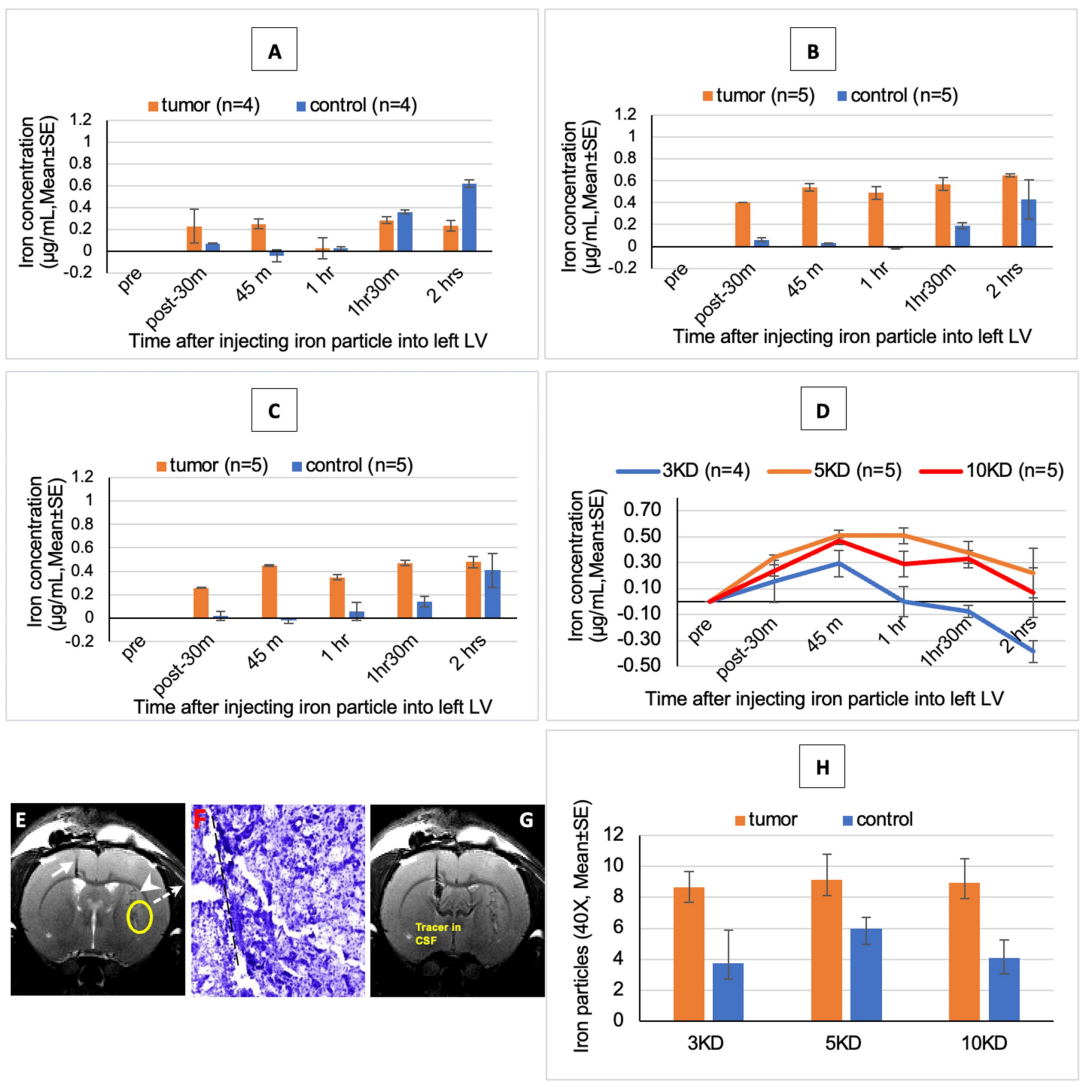
Distribution of varying iron dextran molecular weights in a constant delivery solution (A) Concentration of 3 kD red fluorescent iron dextran tracer in 337 mOsm/L in tumor and normal tissue. (B) Concentration of 5 kD red fluorescent iron dextran tracer in 337 mOsm/L in tumor and normal tissue. (C) Concentration of 3 kD red fluorescent iron dextran tracer in 337 mOsm/L in tumor and normal tissue. (D) Concentration of different sizes of red fluorescent iron dextran tracer in 337 mOsm/L in tumor tissue. (E) T2WI of 10 kD red fluorescent iron dextran (337 mOsm/L). MR image showing the micro-catheter loaded with iron dextran (white arrow), the injecting tumor track (white arrowhead), the spot for measuring iron dextran concentration (yellow circle), and the Nissl staining spot that comes from this area to show tumor histology (dot arrow). (F) Nissl stain (10×) showing the needle track for injecting breast cancer tumor cell (black dot line) and tumor tissue. (G) Image showing iron dextran tracer was injected into the CSF through the left lateral ventricle. (H) Distribution of different sizes of iron dextran tracers with 337 mOsm/L. The 3 kD (A) and 10 kD (C) dextrans distributed preferentially to the tumor in the first 90 minutes with the peak at 45 minutes (D). The 5 kD (B, D) dextran preferentially distributed to the tumor for two hours with the peak between 45 and 60 minutes. T2WI: T2-weighted imaging; MR: magnetic resonance; CSF: cerebrospinal fluid

**Figure 4:**
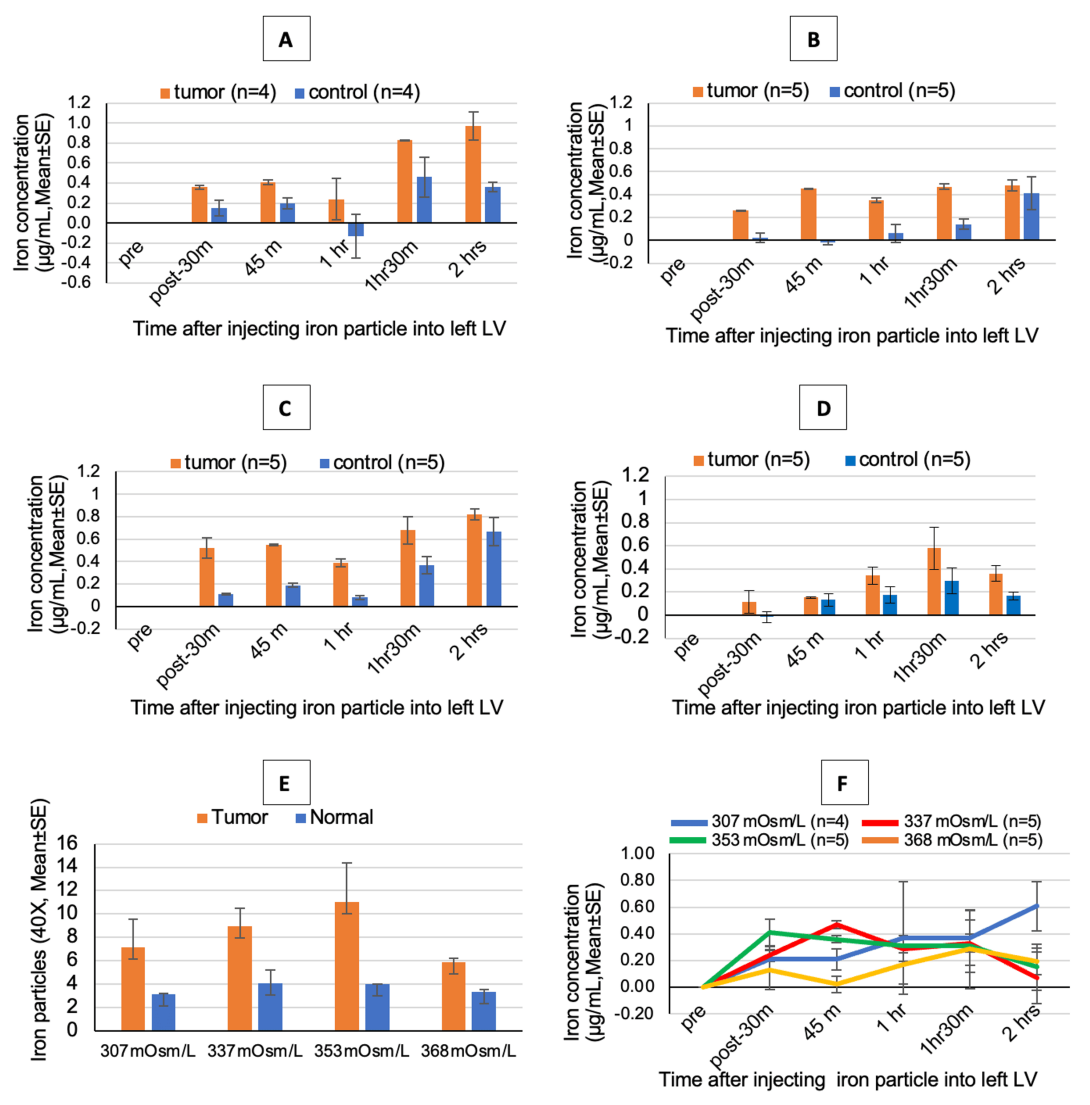
Distribution of 10 kD iron dextran with varying delivery solution osmolarities (A) Concentration of 10 kD red fluorescent iron dextran tracer in 307 mOsm/L in tumor and normal tissue. (B) Concentration of 10 kD red fluorescent iron dextran tracer in 337 mOsm/L in tumor and normal tissue. (C) Concentration of 10 kD red fluorescent iron dextran tracer in 353 mOsm/L in tumor and normal tissue. (D) Concentration of 10 kD red fluorescent iron dextran tracer in 368 mOsm/L in tumor and normal tissue. (E) Distribution of 10 kD dextran tracer in different osmolarity. (F) Concentration of 10 kD red fluorescent iron dextran tracer in different osmolarity in tumor tissue. The higher osmolar injection of 368 mOsm/L did not preferentially distribute to the tumor, and there was equal distribution of the dextrans into the tumor as well as normal tissues (E, F).

The MDA-MB-231 cell solution was successfully injected, and the tumor was successfully implanted in all six groups of rats. We demonstrated that the amount of iron dextran that was distributed in the tumor and normal tissue can be determined effectively following intrathecal injection in micrograms/mL. Iron concentrations determined by the MR technique corroborated with the histopathological determination of the dextran particles (Figure 3H; Figure 4E). This MR technique can be used to determine the amount of drug in the tumor following intrathecal delivery in patients in the future. The measured R2* values correlated linearly with the contrast agent iron concentration with the relationship R2*=10.02×(cFe)+88.028 (R2=0.98) where concentration is given in µg/mL and R2* in s-1 (see Figure 1).

Varying the size of the dextran tracer (at the same osmolarity) changed the preferential distribution of the dextran in the tumor and normal tissues (Figure 3). The 3 kD (Figure 3A) dextran distributed preferentially to the tumor only in the first 45 minutes and the 10 kD (Figure 3C) dextran distributed preferentially to the tumor in the first 90 minutes with the peak at 45 minutes (Figure 3D). The 5 kD (Figure 3B, 3D) dextran preferentially distributed to the tumor for two hours with the peak between 45 minutes and 60 minutes.

Varying the osmolarity of the dextrans infused changed the preferential distribution of the dextran in the tumor and the normal tissues (Figure 4). There was a preferential distribution of the dextrans to the tumor when delivered with an osmolarity of 307, 337, or 353 mOsm/L for 90 minutes following infusion (Figure 4A, 4B, 4C, 4F). Higher osmolar injection at 368 mOsm/L did not preferentially distribute to the tumor, and there was equal distribution of the dextrans into the tumor as well as normal tissue (Figure 4E, 4F).

## Discussion

The delivery of therapeutic drugs to the CNS through intraventricular means is established in the setting of CNS infection and neoplastic processes involving the meninges as well as in the treatment of lymphoma [14-17]. However, the treatment of parenchymal CNS disease has not been well studied. In our prior work, we demonstrated that a drug called DV1 which inhibits tumor growth preferentially accumulated in tumor tissue implanted into mice brains when delivered in a hyperosmolar solution through the ventricle [11]. In this experiment, we used an iron-labeled dextran molecule and hypothesized that a smaller molecular size and higher osmolarity delivery solution would be associated with increased drug delivery to implanted tumor tissue.

Our experiment tested both molecular weight and osmolarity of the delivery solution as independent variables to determine the best combination of size and osmolarity for optimum tumor targeting. Analysis of iron dextran concentration of different molecular sizes reveals two interesting findings. The smallest iron dextran molecule (3 kD) reaches its peak concentration within the tumor tissue earlier than the larger molecules (5 kD or 10 kD). However, the smallest molecule's maximal time of effect, or the time during which the iron dextran is present in the tumor tissue, is shorter than the larger molecules. Comparing the 5 to the 10 kD iron dextran molecule injection with respect to time, it appears as though both have similar time to peak curves.

We then used 10 kD iron dextran as our constant and varied the osmolarity of the delivery solution. The osmolarities used were 307, 337, 353, and 368 mOsm/L. This revealed that the 10 kD iron dextran molecule preferentially accumulated within tumor tissue compared to the normal surrounding brain when delivered in the 307, 337, and 353 mOsm/L delivery solution. When iron dextran was injected into the ventricle in a 368 mOsm/L solution, there was no preferential accumulation in tumor tissue. All three lower osmolarity solutions had reached peak concentrations of iron dextran in tumor tissue within 90 minutes of injection into the lateral ventricle.

While these results show that macromolecules can be passively targeted to tumor tissue in the mouse model, our understanding of CSF homeostasis and handling of macromolecules within the brain limits our ability to understand why this occurs. The classical teaching of CSF physiology is that there is unidirectional flow through the ventricular system and reabsorption of CSF by arachnoid granulations in the subarachnoid space [18,19]. Inherent to this model is the concept of the blood-CSF barrier which consists of the ependymal cells of the ventricle, the choroid plexus epithelium, and the capillary endothelium [8]. However, more recent literature indicates that our understanding of CSF distribution and reabsorption as well as macromolecule permeability is limited.

CSF is mainly composed of water, and in recent literature, the brain has been shown to be permeable to water. Bulat et al. used radiolabeled 3H-water with an inulin molecule control to demonstrate the accumulation of water molecules around and into periventricular capillaries [20]. It has also been shown that aquaporin-4 channels are expressed in astrocytes as well as the ependymal cells lining the ventricles, further supporting that the brain is permeable to water [21,22]. Furthermore, there is evidence of a paravascular pathway of CSF and brain interstitial fluid clearance of macromolecules from the brain through what has been termed the glymphatic system [23].

Macromolecules seem to be handled by the brain in several different ways. At the BBB, there are drug efflux transporters such as P-glycoprotein that serve as an efflux pump to limit the passage of many macromolecules such as chemotherapeutic agents, antibiotics, and opioids from the blood vessels into the brain [13]. The blood-CSF barrier has different characteristics and permeability profiles compared to the BBB, but macromolecule transport occurs at this interface as shown by high expressions of multidrug resistance-associated protein (MRP1) at the choroid plexus epithelium and evidence of active penicillin transport at this interface [8,24-26]. Also, macromolecules from the CSF can diffuse through the brain parenchyma and accumulate along paravascular spaces and are eventually cleared into the systemic circulation [10]. One possible mechanism involved in the clearance of the macromolecules from the paravascular space is through efflux transporters [27].

Based on the data gathered from this experiment, there are two observations that can be made about the optimal conditions under which a dextran macromolecule can be targeted to implanted tumor tissue. The first is that the smallest dextran molecule (3 kDa) is able to reach peak concentrations more quickly than the larger dextran molecules but is cleared from the brain faster. The second is that using a very high osmolarity solution (368 mOsm/L) leads to the non-preferential spread of dextran throughout the brain parenchyma and tumor tissue alike. This demonstrates that very high osmolarity infusion does not provide a therapeutic advantage in the presence of a tumor. However, this finding demonstrates that hyperosmolar drug delivery can be used to target normal cells in the brain such as in the case of antisense oligonucleotide therapy or in antibiotic delivery in infection.

The movements of many different types of macromolecules have been studied in the brain, and each seems to have a different profile of permeability to the brain. Diffusion is the main method by which macromolecules traverse the brain's extracellular matrix, and the diffusion rate is dependent on the distance traversed as well as the molecule's size [8]. Blasberg showed that methotrexate and cytosine arabinoside could cross the ependymal lining into the brain parenchyma when injected into the ventricle but methotrexate did not exchange across brain capillaries while cytosine arabinoside accumulated within neural cells [28]. Penicillin, on the other hand, is eliminated from the CSF through an efflux pump at the choroid plexus and via diffusion across the ependymal surface [26]. In our experiment, we studied the implication of molecular size of the macromolecule dextran but using a different macromolecule may produce a very different result as they would have different lipophilicity and may be subject to different forces such as efflux transporters before reaching the target tissue.

Hyperosmolar solutions have long been a subject of investigation in opening the BBB to deliver therapeutic agents to the CNS. The proposed mechanism is that it temporarily can weaken tight junctions by shrinking the intracellular volume of the endothelial cells of the BBB. However, hyperosmolarity within the CSF has not been elaborated on as a means of delivering therapeutics to the CNS. Given the brain is permeable to water, osmolarity would be a driving factor in CSF homeostasis. We have shown that hyperosmolar solutions delivered directly into the ventricles induce hydrocephalus and that in a state of hydrocephalus, macromolecule clearance can be delayed. This delay in the clearance of macromolecules could be one of the driving factors that allows the said macromolecule, in our case dextran, to diffuse effectively down a concentration gradient into the cells. Further, it could be postulated that the path of least resistance for clearing this macromolecule could be through the vasculature of the tumor tissue via paravascular pathways as the vast majority of tumors tend to be hypervascular.

Limitations

The limitation of this study is that this study purposefully used a single type of molecule with varying molecular weights. There is a limited ability to generalize this to other types of molecules with various charges/sizes. The other limitation is that only one type of tumor model was tested and the results need to be confirmed in other models. 

## Conclusions

Our prior work in addition to this study demonstrated that hyperosmolar intrathecal delivery of drugs preferentially distributes into the tumor as opposed to normal tissues improving the therapeutic index. The distribution of the drugs depends on the size of the molecule as well as the osmolarity of the solution that they are infused. MRI can be used to determine the exact amount of drug delivered to the tumor as well as normal tissues in microgram quantities. In conclusion, hyperosmolar intraventricular delivery of chemotherapeutic drugs could be effective in preferentially delivering drugs to tumors compared to normal tissues.
